# Parotid Masson's tumor: case report^☆^

**DOI:** 10.1016/j.bjorl.2016.01.003

**Published:** 2016-03-29

**Authors:** Filippo Carta, Sara Sionis, Valeria Ledda, Clara Gerosa, Roberto Puxeddu

**Affiliations:** a University of Cagliari, AOU, Policlinico D. Casula, Department of Otorhinolaryngology, Monserrato, Italy; b Department of Pathology, AOU, P.O. S Giovanni di Dio, University of Cagliari, Cagliari, Italy

## Introduction

Masson's tumor, also known as ‘Masson's vegetated intravascular hemangioendothelioma’, is a papillary hyperplasia of the endothelial vascular cells that generally develops in medium-sized veins, but may also be observed in all-sized veins and, less frequently, in arteries.^1^ Pierre Masson in 1923 first described this tumor in inflamed hemorrhoidal plexus and explained its pathogenesis as a primary endothelial proliferation of endothelial cells into the vessel lumen due to obstructive thrombosis, followed by degeneration and necrosis.^2^ Currently, it is considered a reactive vascular proliferation secondary to vascular stasis^3^ that can develop in tendons, head and neck, skin, intracranial, aero-digestive tract, intra-abdominal areas, genital tract, and fallopian canal; it is generally endovascular, but Pins et al.^4^ reported 13 patients presenting with a rare extravascular form. Corio et al. in 1982^5^ reported 14 cases of head and neck Masson's tumor, only one found in the parotid gland; clinical features were not detailed. To the best of our knowledge, this is the first well documented reported case of a Masson's tumor of the parotid gland. The authors want ENT surgeons and pathologists to be aware of this entity.

## Case report

The publication of clinical data with respect of the laws concerning privacy and was approved by the Ethics Committee of the University Hospital, and the patient consented to the publication of the clinical data regarding this case report.

A 43 year-old woman was referred to our Department presenting with a 4 years history of right parotid swelling. The tumor was solid in consistency, had slowly increased in size until 2 cm in its greatest dimension and was mildly painful. The rest of the clinical examination was unremarkable.

**Figure 1:**
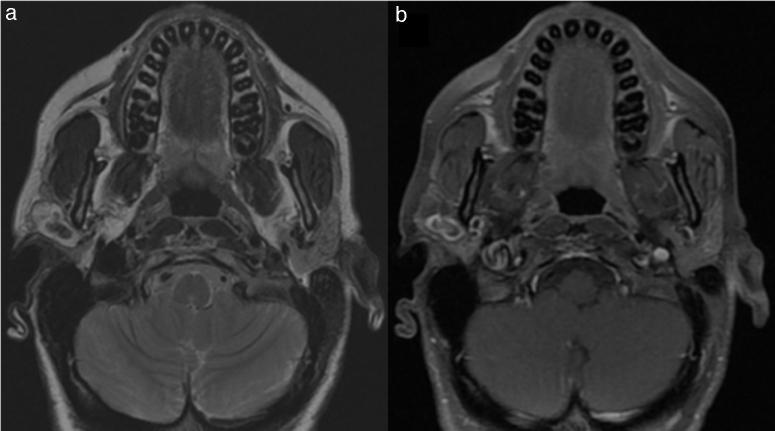
(a) T2-weighted axial sequence shows heterogeneous hyper signal intensity, due to the presence of areas of marked low signal intensity. (b) T1-weighted FAT-SAT axial sequence after medium contrast intake shows an irregular enhancement, since the medium contrast uptake was absent in the central and in the cystic areas, but it was higher in the solid peripheral component of the tumor.

Contrast-enhanced magnetic resonance imaging (MRI) showed a multilobulated lesion of approximately 2.3 cm in diameter within the superficial lobe of the parotid gland. The lesion was elongated and composed of tubular, tortuous and ectasic structures with suggestive characteristics of a hyper vascularized benign lesion (Fig. 1).

**Figure 2:**
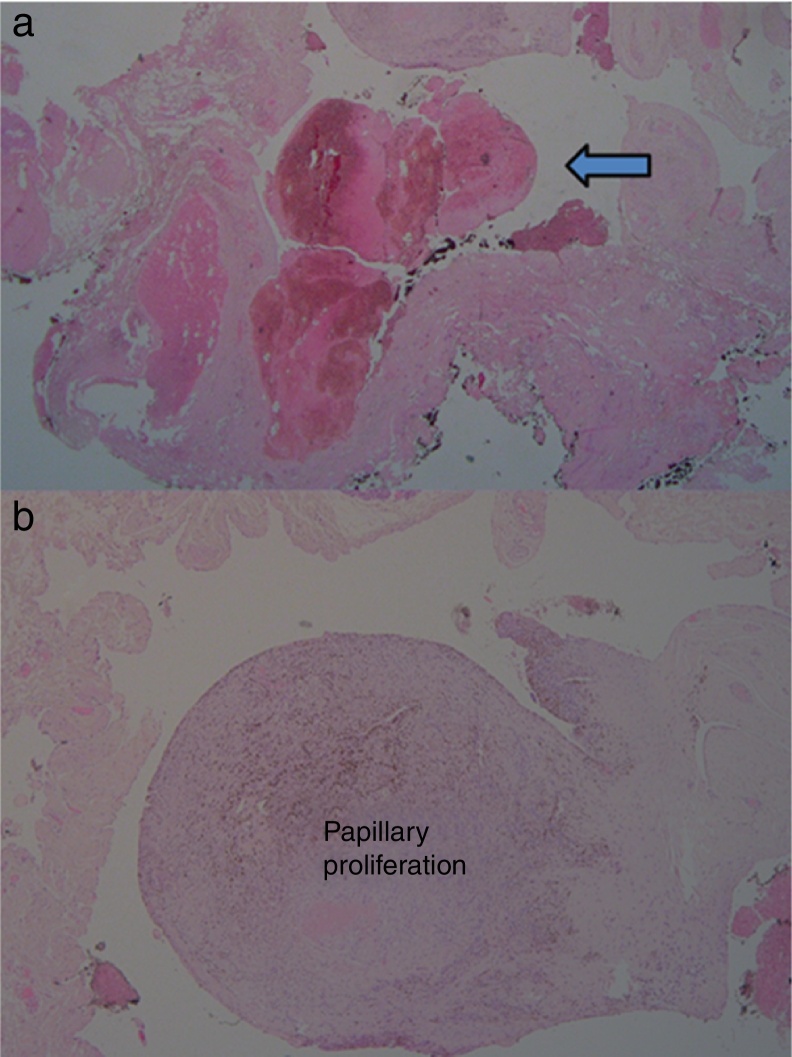
(a) Microscopic examination showed an organizing thrombus (arrow). (b) Microscopic examination showed intravascular papillary proliferation lying of endothelial cells.

Fine needle aspiration cytology was not performed. The patient underwent a subtotal parotidectomy under general anesthesia. The mass was resected within the sovraneural gland, preserving the facial nerve. The removal was performed under microscopic view (Carl Zeiss^®^ – Germany) with a focal length of 250 mm and continuous intraoperative facial nerve monitoring (N.I.M. Response 3.0, Medtronic^®^ – United States). The deeper aspect of the mass was strictly adhering to the peripheral zygomatic branches of the facial nerve and required an extremely precise microscopic dissection to avoid accidental rupture of the lesion and major bleeding. Histological examination showed a major salivary gland (5 cm × 3 cm × 1 cm) with a wide vascular cavity, containing an organizing thrombus and a small papilla protruding into the lumen composed of a single layer of endothelial cells; these cells appeared swollen, without significant pleomorphic or mitotic figures and surrounding a core rich in collagen (Fig. 2). These findings allowed the diagnosis of Masson's tumor. Postoperative outcome was uneventful and the patient was discharged three days after the procedure. After 30 months the patient was well and free from tumor-related symptoms, with an intact facial nerve function.

## Discussion

Masson's tumor is an unusual form of organizing thrombus with excessive papillary proliferation of endothelial cells in normal blood vessels or vascular malformations.^1^ Although the case reported was not related to any predisposing factor, in the literature this lesion is usually related to irradiation and chronic trauma, that may change the venous and arterial flow^3^ causing papillary endothelial proliferation. There is no age predilection (ranging from 9 months to 80 years old) and it occurs equally in males and females, but Chang in 2012^6^ suggested a female predilection mainly due to the tumor's response to estrogens and progesterone.

Masson's tumors are classified in three types: a pure or primary form that arises de novo in dilated vascular spaces, being the most frequent type (56% of cases), a mixed type (secondary or reactive) due to focal changes in a pre-existing vascular lesion (haemangioma, pyogenic granuloma or vascular malformation) and, rarely, a third type resulting from the organization of a hematoma in an extra vascular location.^4^

Head and neck Masson's tumors are found more frequently in the lateral region of the neck, where the most common symptom is a tender or solid slow-growing swelling that can rapidly increase in size, due to the bleeding inside the lesion.^3^ Another frequent site of this lesion is the sino-nasal tract, presenting with recurrent epistaxis.^7^

Parotid Masson's tumors are extremely rare: In 1982, Corio et al. reported a Masson's tumor of the parotid, but no clinical or therapeutic data were detailed in their description^5^; Narwal et al.^8^ described in 2013 an intra-oral Masson's tumor that could be originated from a minor salivary gland but not from the parotid.

Differential diagnosis includes all benign and malignant soft-tissue tumors located in the anatomic region where the Masson's tumor develops; the case reported presented more similarly to typical benign parotid tumors.

Radiologic diagnosis can be challenging: MRI is equivocal in the diagnosis because of the amount of blood vessels and thrombi usually present within the lesion and a radiologic similarity to the low-grade angiosarcoma.^5^ Effectively Masson's tumor typically appears on MRI as minimally heterogeneous on T1-weighted images, mostly isointense to muscle and post contrast T1-weighted images show heterogeneous enhancement that may be suggestive of malignant lesion.^9^ In the present case report, MRI showed T1 heterogeneous hyper intensity, T2 heterogeneous high signal intensity with central low signal intensity and irregular medium contrast uptake in T1 weighted FAT-SAT sequences, more suggestive of a benign hyper vascularized lesion.

Parotid Masson's tumor is a slow-growing benign lesion adhering to the surrounding salivary gland parenchyma; therefore the surgical removal through sub-total or total parotidectomy is considered the gold standard treatment. In our case the main challenge was due to the inflammatory behavior of the lesion, requiring a fine dissection of the branches of the nerve; the use of the operative microscope associated with the intraoperative nerve monitoring allowed the anatomic preservation of all the branches of the facial nerve, avoiding postoperative weakness of the nerve.

Pathological analysis of the case demonstrated an intravascular papillary growth composed of endothelial hyperplasia and a central thrombotic area (Fig. 2). Endothelial proliferation was suspicious for angiosarcoma, but, according to the specific diagnostic features reported by Sarode et al.,^10^ the proliferative process entirely confined to the intravascular space, the regular aspect of the cells of the endothelial layer, and the absence of necrosis and/or cellular atypia in the endothelial proliferation allowed the diagnosis of Masson's tumor.

## Conclusion

The prognosis of Masson's tumor is excellent, with extremely low recurrence rate after complete excision.^3^

## Conflicts of interest

The authors declare no conflicts of interest.
